# Violence against children in sub-Saharan Africa: a call for action

**DOI:** 10.3389/fpubh.2025.1550114

**Published:** 2025-04-10

**Authors:** Enos Moyo, Perseverance Moyo, Hadrian Mangwana, Grant Murewanhema, Tafadzwa Dzinamarira

**Affiliations:** ^1^School of Nursing and Public Health, College of Health Sciences, University of Kwa-Zulu Natal, Durban, South Africa; ^2^Clinical Department, Medical Centre Oshakati, Oshakati, Namibia; ^3^Project Hope Namibia, Windhoek, Namibia; ^4^Department of Adolescent and Women's Health, Faculty of Medicine and Health Sciences, University of Zimbabwe, Harare, Zimbabwe; ^5^Faculty of Health Sciences, School of Health Systems and Public Health, University of Pretoria, Pretoria, South Africa

**Keywords:** violence against children, health effects, risk factors, prevention strategies, challenges

## Introduction

Violence against children (VAC) is recognized as a global public health problem (1). It encompasses all types of violence directed at individuals under 18 years of age, committed by parents, caregivers, peers, romantic partners, or strangers (2). VAC includes all forms of physical and emotional ill-treatment, sexual abuse, neglect, or exploitation that may cause actual or potential harm to a child's health, development, and dignity. Most VAC encompasses at least one of the six primary forms of interpersonal violence: maltreatment, bullying, youth violence, sexual violence, and emotional or psychological violence (2).

Maltreatment encompasses violent punishment and includes physical, sexual, and psychological/emotional violence, as well as neglect of infants, children, and adolescents by parents, caregivers, and other authority figures. Maltreatment occurs primarily in the home but also environments such as schools and orphanages (3). Bullying, encompassing cyberbullying, refers to unwanted aggressive conduct exhibited by an individual or group of individuals who are not siblings or romantically involved with the victim. It encompasses recurrent physical, psychological, or social harm and frequently occurs in schools and other environments where children congregate, including online (2). Youth violence predominantly affects individuals aged 10–29 years, frequently occurring in community environments among acquaintances and strangers. It encompasses bullying and physical assaults, which may involve weapons such as guns and knives, and can also include gang-related activities (4). Intimate partner violence, commonly known as domestic violence, encompasses physical, sexual, and emotional abuse perpetrated by an intimate partner or former partner. While males may also experience victimization, intimate partner violence predominantly impacts females. It frequently occurs among girls in child marriages and early or forced marriages (5).

Sexual violence encompasses non-consensual completed or attempted sexual contact, as well as acts of a sexual nature that do not involve physical contact, including voyeurism and sexual harassment. It also includes acts of sexual trafficking against individuals unable to consent or refuse, along with online exploitation (3). Emotional or psychological violence consists of the restriction of a child's movements, denigration, ridicule, threats, intimidation, discrimination, rejection, and other non-physical forms of hostile treatment (2). Violence directed at individuals based on their biological sex or gender identity can be classified as gender-based violence (6).

It is estimated that approximately one billion children aged 2–17 years worldwide have encountered physical, sexual, or emotional violence or neglect (7). A study in four sub-Saharan African countries found that 20–37% of girls encounter sexual violence, while 12–25% face physical violence before turning 18 years old (8). Ten percent of boys experience sexual violence, while 30 to 45 percent experience physical violence (8). Progress has been made in decreasing the incidence of child abuse in Sub-Saharan Africa (SSA). From 2010 to 2019 in Kenya, there was a reduction of over four million children subjected to sexual violence, a 21% decrease in the prevalence of physical violence among males, and a significant decline in emotional violence experienced by females at the hands of caregivers (9). In Eswatini, from 2007 to 2022, ~170,000 fewer children experienced sexual violence (9). Despite observed progress in the region, further efforts are required to guarantee that all children are free from any form of violence. In this perspective article, we highlight the health effects of VAC and its associated risk factors while proposing interventions that have demonstrated effectiveness in certain countries within SSA for preventing VAC. By underscoring the health implications of VAC, we aim to instill a sense of urgency among governments and non-governmental organizations in SSA to address this issue. Additionally, identifying the risk factors associated with VAC may aid in developing targeted prevention programs. Stakeholders in SSA may consider prioritizing the suggested interventions, given their successful implementation in select countries.

## Methodology

In this perspective article, we conducted a literature search to inform our understanding of VAC and its health impacts, focusing on sub-Saharan Africa. Rather than a systematic or scoping review, the objective was to provide a comprehensive viewpoint supported by existing literature. We searched for peer-reviewed articles published in English between 2000 and 2024 using the Google Scholar, ScienceDirect, and PubMed databases. The terms employed for the literature search included “violence against children,” “health effects,” “sub-Saharan Africa,” “risk factors,” “prevention strategies,” and all countries within SSA. Boolean operators “AND” and “OR” were employed to obtain articles containing both terms or either term. Wildcard and truncation symbols were used to broaden a search term, encompassing all variations of a root word. All full-text versions of articles that potentially offered relevant information regarding our objectives were obtained and assessed. The reference lists of all retrieved articles were examined for additional relevant articles not identified through database searches. Two authors (EM and PM) independently assessed the titles and abstracts of all identified articles and subsequently compared their findings. Discrepancies were addressed through discussion or adjudication by a third author (TD). Two authors independently retrieved relevant information from the selected studies and subsequently compared their findings before presenting them in narrative form.

## Health effects of violence against children

Violence against children results in both direct and indirect health consequences for the victims. Direct health effects can encompass severe injuries that may result in disability or mortality (2). Early exposure to violence can adversely affect brain development and harm various systems, including the nervous, endocrine, circulatory, musculoskeletal, reproductive, respiratory, and immune systems, leading to lifelong repercussions (10). VAC adversely impacts cognitive development, leading to educational and vocational underachievement (11). The indirect health effects of VAC arise from repeated exposure to or observation of violence, leading to heightened activation of neuroendocrine, autonomic, immunological, and neuropsychological systems. The activation leads to persistent stress in children, as indicated by elevated heart rate, hypervigilance, and cortisol levels (10). Chronic stress and fear can adversely affect mental health and coping mechanisms, leading to issues such as depression, substance abuse (12), suicide (13), impaired learning (11), socialization (14), and productivity (15). Stress reactions can become normalized, leading individuals to respond aggressively to others or interpret aggression in others even without a real threat. The enduring impacts of VAC on cognitive and psychosocial development encompass impaired attachment, failure to thrive, trauma, anxiety, depression, and antisocial and self-destructive behaviors (16). Self-destructive behavior can include risky sexual practices, leading to unintended pregnancies, and sexually transmitted infections (12). VAC is associated with the later development of non-communicable diseases, including cardiovascular disease, cancer, chronic lung disease, and diabetes (17). VAC elevates the likelihood of revictimization or perpetration in the later stages of life. Research indicates that girls face a heightened risk of sexual assault and intimate partner violence, whereas boys are more frequently identified as perpetrators (18, 19).

## Risk factors of violence against children in SSA

Previous studies conducted in SSA identified several risk factors associated with VAC. These risk factors can be divided into individual, close-relationship, and societal factors. Risk factors at the individual level include age, being orphaned before the age of 13 years, experiencing emotional abuse before the age of 13 years (20), exposure to drugs, alcohol, crime, conflict, and disability (21). Conflicting evidence exists regarding the impact of age on VAC. One study in Zimbabwe identified older age as a risk factor for VAC (12), while another study in Burkina Faso reported younger age as a risk factor (21). Research indicates that younger children are more likely to encounter violent disciplinary practices both at home and in educational settings, in contrast to older adolescents who are not enrolled in school (21). The elevated risk of VAC among individuals with disabilities may stem from heightened parental stress, challenging parent-child dynamics, and familial conflict in households with a disabled child (22).

Close-relationship factors include having lived with three or more families during childhood (20), sharing a household with a depressed individual (21), living in households without either parent (15), experiencing poor family relationships (12), the presence of adult illness in the home (12), having peer relationships (12), and encountering unsupportive teachers at school (12). The HIV epidemic in Africa has led to numerous parental deaths, resulting in a significant number of orphaned children. The deaths disintegrated family structures, potentially elucidating the correlation between VAC and having resided with three or more families during childhood, as non-parental caregivers may exhibit less tolerance toward children's behavior compared to biological parents (23). The presence of an ill adult in the household may contribute to frustrations that could result in violent behavior toward children. Teachers lacking compassion may deliberately cause physical pain to discourage undesirable behavior in students (12).

Societal risk factors for VAC include increased exposure to community members engaged in substance abuse and criminal activities, as well as low family socioeconomic status (12, 15). Individuals within the community who engage in substance use frequently exhibit intoxication, which can result in the abuse of children. Certain community members may introduce children to substance abuse, potentially leading to the children being subjected to abuse for displaying unacceptable behavior (15). Low socioeconomic status among parents or caregivers can contribute to stress and elevate the risk of VAC (12).

## A critical analysis of strategies to prevent violence against children in SSA

The prevention of VAC necessitates a multifaceted strategy. Successful strategies across various contexts encompass the implementation and enforcement of laws, the establishment of norms and values, the creation of a safe environment, support from parents and caregivers, economic strengthening, provision of response and support services, and the promotion of education and life skills (24).

Despite the established effectiveness of these strategies, several challenges persist in the implementation across SSA. One key challenge is governments' lack of political will and prioritization of child protection issues, often due to competing national priorities, political instability, or insufficient public awareness of VAC's long-term societal consequences. In many SSA countries, there is also inadequate institutional capacity and resources to enforce existing laws, which hampers their effectiveness in preventing violence against children. For example, while laws may criminalize child abuse, implementation remains weak due to inadequate infrastructure, poor law enforcement, and limited training of relevant personnel such as law enforcement and healthcare workers.

Additionally, the establishment of norms and values that protect children is complicated by deep-rooted socio-cultural practices, such as corporal punishment being viewed as a standard disciplinary measure in many households. This cultural acceptance of violence makes it difficult for educational campaigns or laws to change behavior effectively. Efforts to create a safe environment are often stymied by inadequate community engagement and a lack of local ownership in child protection efforts. In some areas, communities may resist or remain unaware of the value of such strategies due to entrenched traditions, limited media coverage, or a lack of community-based programs that provide practical solutions.

Laws may restrict violent punishment, criminalize the sexual abuse and exploitation of children, and regulate youth access to weapons (25). However, despite the existence of these laws, implementation remains inconsistent, particularly in rural and conflict-affected regions, where governmental oversight and judicial systems are weaker. In some countries, the focus on lawmaking has outpaced the resources required to ensure these laws are fully implemented and enforced. Furthermore, the low capacity of child protection systems often results in inadequate follow-up, leading to minimal impact on the ground.

A secure home environment can mitigate VAC. A program conducted in the Democratic Republic of Congo (DRC) aimed at enhancing overall family wellbeing and preventing violence, including intimate partner violence and harsh discipline toward children, resulted in a decrease in the application of physical and emotionally harsh discipline within the intervention group (26). The program focused on disseminating information regarding the causes and consequences of violence against women and children, as well as imparting skills in stress management, psychosocial support, and positive parenting strategies (26). However, such programs are not universally implemented, and many caregivers in SSA lack access to such resources due to geographic, economic, or logistical barriers. Moreover, socioeconomic stressors, such as poverty and unemployment, can undermine the effectiveness of family-oriented programs, making it more difficult for parents and caregivers to engage with and benefit from these interventions.

Support for parents, children, and caregivers can be provided through various strategies. These include promoting responsive parenting, reducing family conflict, and enhancing caregivers' capabilities to access and navigate available resources (27). While these strategies have shown promise, many communities in SSA face significant barriers, such as low literacy levels, limited access to counseling, and a lack of support networks, which hinder the effectiveness of these interventions. A program implemented in Rwanda that utilized parent-child and caregiver support strategies resulted in enhanced parent-child relationships, diminished harsh discipline, and lowered levels of caregiver depression and anxiety (28). However, the scalability of such programs across SSA remains a challenge, as there is a lack of funding for nationwide implementation and cultural adaptability in different contexts.

Additional strategies for supporting parents and caregivers include educating them about child development, informing them of the risks associated with physical punishment, raising awareness of non-violent disciplinary methods, and providing practical guidance for addressing child misbehavior (29). Despite these efforts, the widespread acceptance of corporal punishment and a lack of educational resources means that many parents are either unaware of the consequences of their actions or lack the skills to implement non-violent discipline effectively. A program in Nigeria aimed at decreasing reliance on corporal punishment and educating parents on effective child management techniques led to a decline in parental reports of physical violence in child training (30). Educating parents on effective communication with children, empathetic listening, establishing routines and household rules, self-care practices, and stress management strategies may reduce VAC (31). A study in Liberia focusing on diminishing harsh discipline, promoting positive parenting, and enhancing parent-child interactions achieved a nearly 56% reduction in caregiver-reported harsh punishment practices, an increase in positive behavior management strategies, and improved caregiver-child interactions (32).

Strategies for income and economic strengthening aimed at reducing VAC in SSA include the formation of saving groups and training, provision of credit access to parents and youth at reasonable interest rates, livelihood planning training, provision of seed capital grants to initiate or expand livelihood activities, and individualized mentoring and coaching on livelihood development (25). A study in West Africa incorporating an economic intervention and family support component reported a decrease in harsh discipline, an increase in supportive parenting attitudes, improved child-parent relationship quality, and a reduced likelihood of children experiencing physical and emotional violence at home (33).

Improving mental health and psychosocial outcomes is essential for reducing VAC among individuals who have experienced trauma. Strategies include psychoeducation, training in relaxation techniques, encouraging the identification of major family issues, conflict resolution training in communities, and effective parenting training (25). A study in the DRC providing a family-focused, psychosocial intervention to war-exposed youths demonstrated that those in the intervention group exhibited reduced post-traumatic stress symptoms at 3 months. Additionally, caregivers noted a moderate to considerable decrease in conduct problems among these youths (34).

Enhancing essential life skills, including communication, relationship development, and awareness of gender-based violence and sexual and reproductive health, can contribute to the reduction of VAC (25). Life skills may also be provided to parents or caregivers, encompassing communication skills, education on recognizing violence and abuse, and strategies for supporting adolescent girls (27). A study in Ethiopia among Sudanese and South Sudanese refugees aimed at reducing gender-based violence and empowering adolescent girls in a humanitarian context found a decrease in reported child marriages within the intervention group (35). While life skills programs have had positive outcomes in some contexts, their reach remains limited, and many young people, especially in rural or conflict-affected areas, do not have access to such programs. Furthermore, gender norms and power dynamics in some SSA countries limit the effectiveness of these programs, particularly those aimed at empowering adolescent girls or addressing gender-based violence.

## Conclusion

In conclusion, despite evidence-based strategies for preventing VAC in SSA, significant gaps in implementation, cultural resistance, and limited resources continue to hinder the effectiveness of these strategies. To address these challenges, governments and NGOs must focus on designing interventions and building the institutional and community capacity to implement and sustain these strategies effectively. There is an urgent need for multi-sectoral approaches that integrate legal, cultural, social, and economic reforms to ensure the protection of children and the reduction of VAC.
